# Visualization of gadolinium transport across the blood-brain barrier along perivascular clearance pathways

**DOI:** 10.1186/s41747-025-00672-0

**Published:** 2026-02-04

**Authors:** Svea Seehafer, Yvonne Mrosek, Lars-Patrick Schmill, Schekeb Aludin, Olav Jansen, Carl Alexander Gless, Johanna Rümenapp, Naomi Larsen

**Affiliations:** 1 Department of Radiology and Neuroradiology, University Hospital of Schleswig-Holstein, Kiel, Germany; 2 Division of Neurological Pain Research and Therapy, Clinic for Neurology, University Hospital of Schleswig-Holstein, Kiel, Germany

**Keywords:** Angiography (digital subtraction), Blood-brain barrier, Contrast media, Glymphatic system, Magnetic resonance imaging

## Abstract

**Objective:**

We investigated the transport of gadolinium-based contrast agent (GBCA) across the blood-brain barrier (BBB) along the perivascular spaces as part of the glymphatic drainage in patients with iatrogenic BBB disruption following digital subtraction angiography (DSA).

**Materials and methods:**

A retrospective analysis was conducted on patients who underwent DSA for diagnosis and/or treatment of intracranial aneurysms and received a 3-T magnetic resonance imaging (MRI) within the following day. Exclusion criteria included states with a suggested impairment of BBB integrity, such as neurodegenerative diseases or suspected glymphatic impairment. BBB disruption was assessed using a pre- and post-contrast three-dimensional T1-weighted volume-isotropic turbo spin-echo sequence. Patterns of GBCA distributions were described. The localization of GBCA-extravasation was correlated with perivascular spaces visualized on the coregistered T2-weighted sequences. Fisher’s exact test and logistic regression were used.

**Results:**

Out of 43 patients, 30 (69.8%) exhibited visible BBB disruption. BBB disruption was significantly more often observed after therapeutic DSA (*p* = 0.004). GBCA-enhancement patterns indicated a localized pial enhancement in 96.7% of affected patients, with additional parenchymal enhancement along the perivascular spaces in 56.7%. Enhancement was predominantly located in the downstream territories of probed vessels, suggesting a potential association with glymphatic transport. An illustrative case with serial MRI examinations is presented, demonstrating time-dependent GBCA-enhancement patterns.

**Conclusion:**

The study provides *in vivo* evidence of GBCA transport patterns following iatrogenic BBB disruption, which may correspond to parts of the proposed glymphatic pathways. Our results indicate a sequential progression of contrast enhancement, initially manifesting at the brain surface and subsequently extending along perivascular spaces to the subarachnoid space.

**Relevance statement:**

Understanding BBB disruption and glymphatic transport with MRI imaging methods may improve neurovascular disease management.

**Key Points:**

- BBB disruption post-DSA may facilitate GBCA transport via glymphatic pathways, offering novel and hypothesis-generating insights into brain clearance mechanisms.
- GBCA enhancement followed a chronological and spatial pattern, suggesting an organized cerebrospinal-interstitial exchange system relevant for brain clearance.
- Findings highlight potential implications for BBB integrity in neurovascular health with prospective implications for diagnostic imaging.

**Graphical Abstract:**

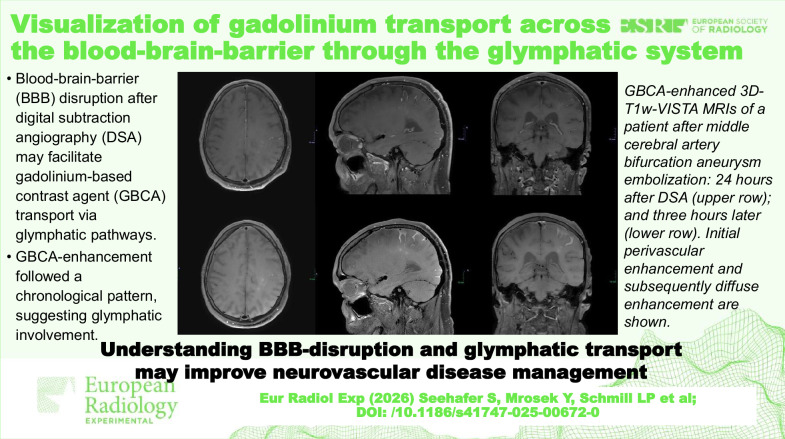

## Background

It is hypothesized that exchange between cerebrospinal fluid (CSF) and interstitial fluid (ISF) of the central nervous system (CNS) is complementarily regulated by the blood-brain barrier (BBB) and the so-called glial-lymphatic (glymphatic) system. The latter was first described in rodents by Iliff et al [1] who postulated an exchange system of CSF and ISF, regulated by aquaporin-4 water channels on astrocyte end feet and driven by bulk flow. When superficial arteries from the surface penetrate into the parenchyma, they do so with adjacent perivascular spaces (PVS). They are filled with CSF-like fluid and enclosed by the BBB on one side and astrocyte end feet on the other side, which in some references are also considered part of the BBB [2]. The water from the CSF enters the ISF through the aquaporin-4 channels as well as the little gaps between the astrocyte end-foot processes. The ISF then flows through the interstitial space, transporting solutes and metabolic waste products, toward the perivenular PVS and is subsequently cleared into the subarachnoid compartment or along the walls of draining veins and sinus to the deep cervical lymph nodes [3]. Also, the subarachnoid space is believed to be compartmentalized. Recently, a fourth meningeal membrane, the subarachnoid lymphatic-like membrane (SLYM), was postulated. It is located within the subarachnoid space between the arachnoid and pia, surrounds the vasculature and was observed to retain tracers. It is supposed to facilitate the peri-arterial influx of CSF and to support the unidirectional glymphatic-CSF transport [4].

It is believed that BBB disruption and glymphatic dysfunction interact and both might provoke neurodegenerative diseases [5]. However, the existence of such a system in humans and the driving forces are still under debate [6]. Additionally, alternative drainage pathways are suggested and discussed in the literature. One of which is the intramural peri-arterial drainage (IPAD) pathway that suggests that tracers injected into the CSF enter and leave the brain along separate peri-arterial basement membrane pathways [7, 8].

Several imaging approaches aim to investigate various aspects of ISF-drainage pathways, involving both the components of the glymphatic system and the IPAD pathway. Some studies revealed an enhancement in predefined locations in the brain parenchyma, the subarachnoid and intraventricular space and inside the superior sagittal sinus in serial MRI scans after intrathecal gadobutrol administration [9]. Especially the inability of conventional MRI contrast agents to cross an intact BBB makes it hard to assess its function *in vivo*, also because contrast-enhanced images are mostly acquired shortly after contrast agent injection, leading to an accumulation in areas with BBB disruptions. Yet, it is generally accepted that gadolinium-based contrast agent (GBCA) after intravenous administration gradually accumulates in the CSF in delayed MRI acquisitions with heavily T2-weighted fluid-attenuated-inversion-recovery sequences despite an intact BBB [10]. Current imaging approaches therefore focus on the detection of contrast agents in the human brain with those sequences that are highly sensitive to low GBCA concentrations in the CSF and PVS of the basal ganglia [11]. However, spatial resolution is low, hampering especially the visualization of PVS. Three-dimensional (3D) T1-weighted turbo spin-echo sequences, so-called black-blood sequences, are also known to be very sensitive in contrast enhancement (CE) detection and are shown to be able to detect more brain lesions in different pathological conditions [12–14].

Reversible iatrogenic BBB disruptions are thought to improve therapeutic delivery to the CNS. An example of such a technique is the MRI-guided transcranial focused ultrasound [15, 16]. Studies with subsequent MRI demonstrated contrast accumulation in the adjacent structures [17].

Iodinated contrast agents, used for computed tomography (CT) and digital subtraction angiography (DSA), are known to cause local reversible BBB disruptions [18–20]. However, the patterns of GBCA transport and drainage have not yet been visualized since they have been mainly described in case reports [15, 17] and postinterventional CT examinations [19, 21, 22] with insufficient spatial resolution, especially for the brain parenchyma.

Taking advantage of this phenomenon, this study aimed to investigate and visualize in humans the transport of GBCA across the BBB through the parenchyma, hypothetically corresponding to the proposed ISF-drainage pathways, by using the aforementioned so-called T1-weighted black-blood sequence in patients with iatrogenic BBB disruption.

## Materials and methods

### Patients

This retrospective study included patients who underwent DSA from 2019 to 2024 for diagnostic or treatment purposes of intracranial aneurysm and received a 3-T MRI including a 3D T1-weighted black-blood sequence within the following day after DSA at the same institution. All patients provided a signed informed broad consent allowing the use of images and clinical data for research. Approval of the institutional review board was obtained (registry number: D 510/24).

**Fig. 1:**
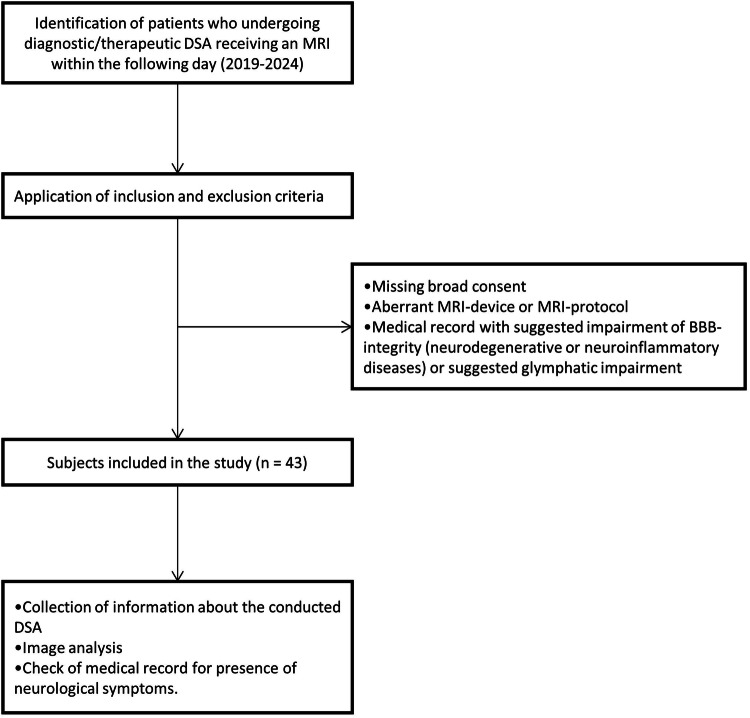
Flowchart of inclusion and exclusion criteria

For further analysis, information about the conducted DSA was collected with regard to duration defined as the interval between the first and last series saved in the local Picture Archiving and Communication System, probed vessels according to the report, amount of injected iodine-based contrast agent (Imeron® 300, 300 mg/mL, Bracco Imaging/Ultravist® 300, 300 mg/mL, Bayer Vital, depending on availability) defined as number of saved series multiplied by 7 mL (mean amount for one series due to dilution) and type of intervention. The digital medical record was checked for the presence of neurological symptoms after the DSA. Exclusion criteria comprised states with a suggested impairment of BBB integrity, such as neurodegenerative diseases like Alzheimer’s disease or neuroinflammatory conditions like multiple sclerosis. Also, patients with suggested glymphatic impairment, such as patients with idiopathic normal pressure hydrocephalus, were not included in the study (Fig. 1).

### MRI protocol

MRI data were obtained from the same device (Ingenia CX 3 T, Philips), equipped with a 32-channel receiver coil. The imaging protocol consisted of a diffusion-weighted imaging (DWI) sequence, an axial T2-weighted turbo spin-echo sequence, as well as a time-of-flight and 3D volume-isotropic turbo spin-echo acquisition (VISTA), called black-blood sequence, before and after administration of weight-adapted intravenous GBCA respectively Gadovist® 1.0 mmol/mL, Bayer Vital, 0.075 mL per kg body weight, with a delay of exactly 5:36 min:s. This was the duration of the time-of-flight sequence, which is acquired as a waiting sequence immediately after the intravenous contrast administration. Sequence parameters are listed in Table 1. We routinely employ this MRI protocol in patients with incidental intracranial aneurysms for risk stratification, after embolization of an intracranial aneurysm as a baseline for follow-up, and in patients with a new neurological deficit after endovascular treatment. The same sequence protocol was used for all patients.

**Table 1:** MRI sequence parameters

Philips Ingenia CX 3 T	DWI	T2	TOF	T1w_VISTA_BB
Echo time	72 ms	80 ms	5.8 ms	27 ms
Repetition time	4,112 ms	5,986 ms	25 ms	700 ms
Flip angle	90°	90°	20°	90°
Matrix	288 × 288	1120 × 1120	864 × 864	640 × 640
Field of view	100 mm	79.29 mm	100 mm	125 mm
Orientation	Axial	Axial	Axial	Sagittal
Duration	00:49 min	03:36 min	05:36 min	05:12 min

*ms* Milliseconds, *mm* Millimeters, *min* Minutes

### Image analysis

Images were assessed by two radiologists (3 years of experience) and one neuroradiologist (15 years of experience). In case of discrepancies, a consensus reading was performed. First, the presence of a BBB disruption (hyperintense signal on post-contrast black-blood images without a correlate on unenhanced images) with localization in the brain parenchyma, pial, or subarachnoid space was analyzed. Here, “pial” was defined as localized CE along the brain surface. “Parenchymal” was defined as CE within the brain parenchyma. “Subarachnoid” was defined as diffuse CE in the subarachnoid space. If present, this was spatially correlated with a vessel territory with the help of the time-of-flight sequence and checked if this territory was probed during DSA. In case of a parenchymal CE, the correlation with PVS was checked with the aid of the axial T2-weighted sequence after image coregistration. The DWI sequence was examined for the presence of diffusion restrictions resulting from acute embolic infarctions.

### Statistical analysis

Descriptive statistics were used for CE pattern description. Categorial data are presented as counts and percentages, and continuous data are presented as means and ranges with standard deviation. A relationship between BBB disruption and diagnostic or therapeutic DSA was analyzed with Fisher’s exact test. The association between the presence of BBB disruption and the DSA duration and time between DSA and MRI was analyzed using a logistic regression analysis. Association with sex was analyzed with Fisher’s exact test and with age using logistic regression. Finally, the association between BBB disruption and the presence of embolism was analyzed with Fisher’s exact test. All analyses were carried out using GraphPad Prism (version 10.4.0), and *p*-values lower than 0.05 were considered significant.

## Results

### Patient demographics

In total, 43 patients were recruited for this study, of which 32 (74.4%) were female, and 11 (25.6%) were male. Age was 58.6 ± 11.8 years (mean ± standard deviation); 17 patients underwent DSA for diagnostic purposes, 26 for treatment purposes. DSA duration was 53 ± 38.9 min (mean ± standard deviation). The mean time between DSA and MRI examination was 15.7 ± 9.9 h (mean ± standard deviation). Patient data are summarized in Table 2.

**Table 2:** Patient demographics

	*N* = 43
Basic demographics	
Biological sex	
Female	32 (74)
Male	11 (26)
Age (years)	
Mean	58.6
Standard deviation	11.82
Minimum	37
Maximum	82
DSA	
Purpose	
Diagnostic	17 (40)
Panangiography	12
Focussed DSA	5
Therapeutic	26 (60)
Web device	11
Contour device	4
Stenting	4
Stenting and coiling	3
Contour device and coiling	2
Coiling	1
Web device, stenting and coiling	1
Duration (min)	
Mean	52.95
Standard deviation	38.91
Minimum	7
Maximum	180
Estimated iodine contrast agent injection (milliliters)	
Mean	96.7
Standard deviation	40.77
Minimum	28
Maximum	217
Time between DSA and MRI (h)	
Mean	15.65
Standard deviation	9.92
Minimum	1
Maximum	26

Values *n* (%) unless otherwise stated

#### Detection of BBB disruption as CE and its relation with DSA

Of all 43 patients, 30 patients (69.8%) presented a BBB disruption defined as a contrast enhancement. At subgroup analysis, 7 of 17 patients (41.2%) who received a diagnostic DSA and 23 of 26 patients (88.5%) who underwent a therapeutic DSA had a BBB disruption. No association with sex (*p* = 0.061) or age (*p* = 0.191) was found. However, a significant association was found between the intervention type and the presence of BBB disruption, with significantly more CE after therapeutic DSA (*p* = 0.035). Yet, both the total intervention duration and the amount of injected iodine-based contrast agent were not significantly associated with the presence of CE. Furthermore, a significant positive association for time between DSA and MRI with the detection of BBB disruption (*p* = 0.026) was observed. In case of observed BBB disruption, a significant association with the presence of microembolism, defined as DWI lesions, was seen (*p* = 0.003). Results of the statistical analysis are summarized in Table 3. In 9 patients, symptoms after DSA were documented, 8 of whom had a BBB disruption. The symptoms of 6 patients did not exceed headache and nausea and did not include any focal neurological deficits. Two patients developed focal neurological deficits, one of which showed a left-sided multimodal neglect, and one had a weakness in the right arm. These symptoms could in both cases be explained by the presence and localization of embolism in the according brain areas. The other patient had a reduced level of consciousness on the second postinterventional day. This patient also received a follow-up MRI showing multiple embolisms.

**Table 3:** Results of statistical analysis

Parameters	*p*-value
Biological sex and the presence of BBB disruption	0.0612*
Age and presence of BBB disruption	0.1905**
Intervention type and presence of BBB disruption	0.0035*
Intervention duration and presence of BBB disruption	0.1905**
Injected iodine-based contrast agent and the presence of BBB disruption	0.5361**
Time between DSA and MRI and presence of BBB disruption	0.0258**
Presence of BBB disruption and presence of microembolism	0.0028*

* Fisher’s exact test

** Logistic regression

#### Pattern of CE

**Fig. 2:**
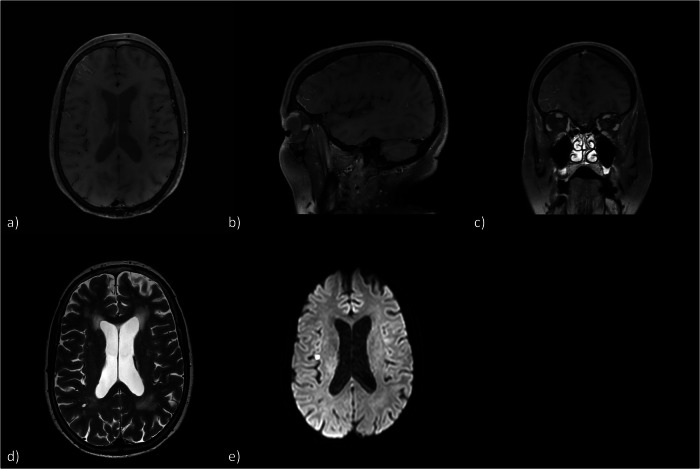
MRI examination 24 h after digital subtraction angiography for aneurysm embolization of the right MCA bifurcation with a WEB device. **a** Axial reconstruction of three-dimensional T1-weighted sequence 5 min after contrast administration, showing a pial enhancement in the right middle cerebral artery territory; sagittal (**b**) and coronal (**c**) reconstructions of the same sequence. **d** Axial T2-weighted sequence on the same level of **a**. **e** DWI sequence showing microembolism in the right insular region (one of seven DWI lesions in total, five in the MCA territory and two in the PCA territory). MCA, Middle cerebral artery; DWI, Diffusion-weighted imaging; MRI, Magnetic resonance imaging; PCA, Posterior cerebral artery; WEB, Woven EndoBridge

**Fig. 3:**
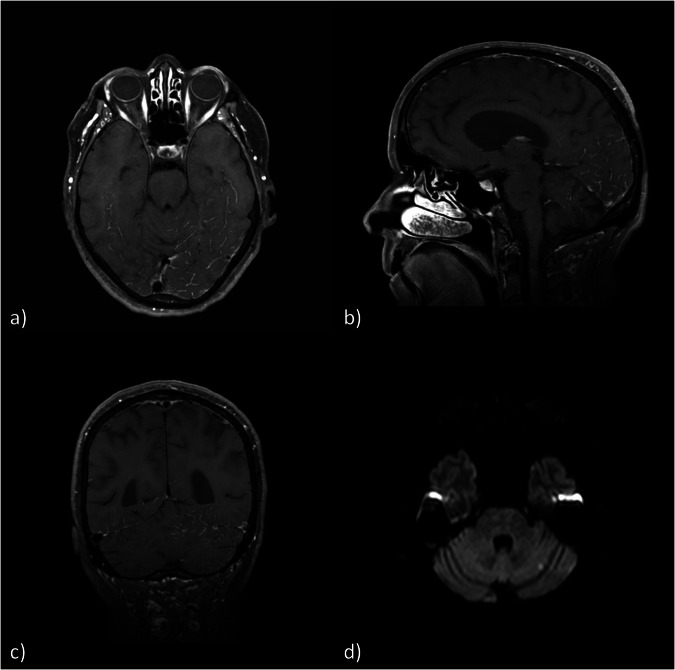
MRI examination 3 h after diagnostic panangiography. This patient was not included in the statistical analysis as the images were acquired from a different MRI device (Philips Achieva 3 T, Best, The Netherlands). **a** Axial reconstruction of three-dimensional T1-weighted sequence 5 min after contrast administration showing a pial enhancement in the posterior cerebral artery territory; sagittal (**b**) and coronal (**c**) reconstructions of the same sequence. **d** DWI sequence showing the only present microembolisms in the left SUCA territory. DWI, Diffusion-weighted imaging; MRI, Magnetic resonance imaging; SUCA, Superior cerebrallar artery

**Fig. 4:**
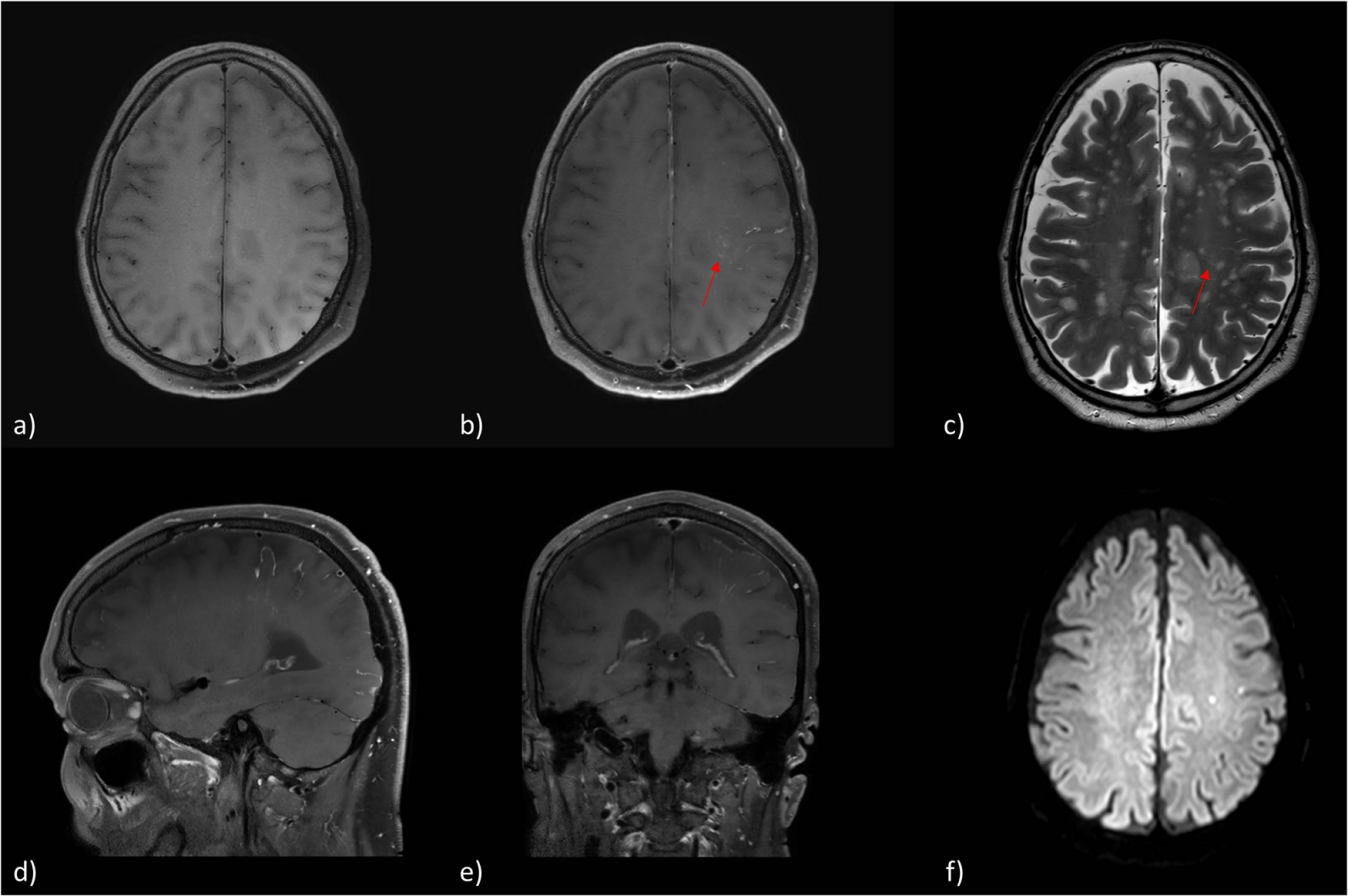
MRI examination 24 h after digital subtraction angiography for aneurysm embolization of the left MCA bifurcation with a Woven EndoBridge device. **a** Axial reconstruction of native three-dimensional T1-weighted sequence before GBCA administration. **b** Axial reconstruction of three-dimensional T1-weighted sequence 5 min after GBCA administration showing a pial and parenchymal enhancement (red arrow) in the left perirolandic region. The pial enhancement is limited to the immediate surrounding of the cerebral vasculature; **c** axial T2w sequence on the same level depicting the course of the perivascular spaces of the centrum semiovale (red arrow); sagittal (**d**) and coronal (**e**) reconstruction of the three-dimensional T1-weighted sequence 5 min after GBCA administration. **f** DWI sequence showing the only two present microembolisms in the left hemisphere, including the precentral gyrus. MCA, Middle cerebral artery; DWI, Diffusion-weighted imaging; GBCA, Gadolinium-based contrast agent; MRI, Magnetic resonance imaging

**Fig. 5:**
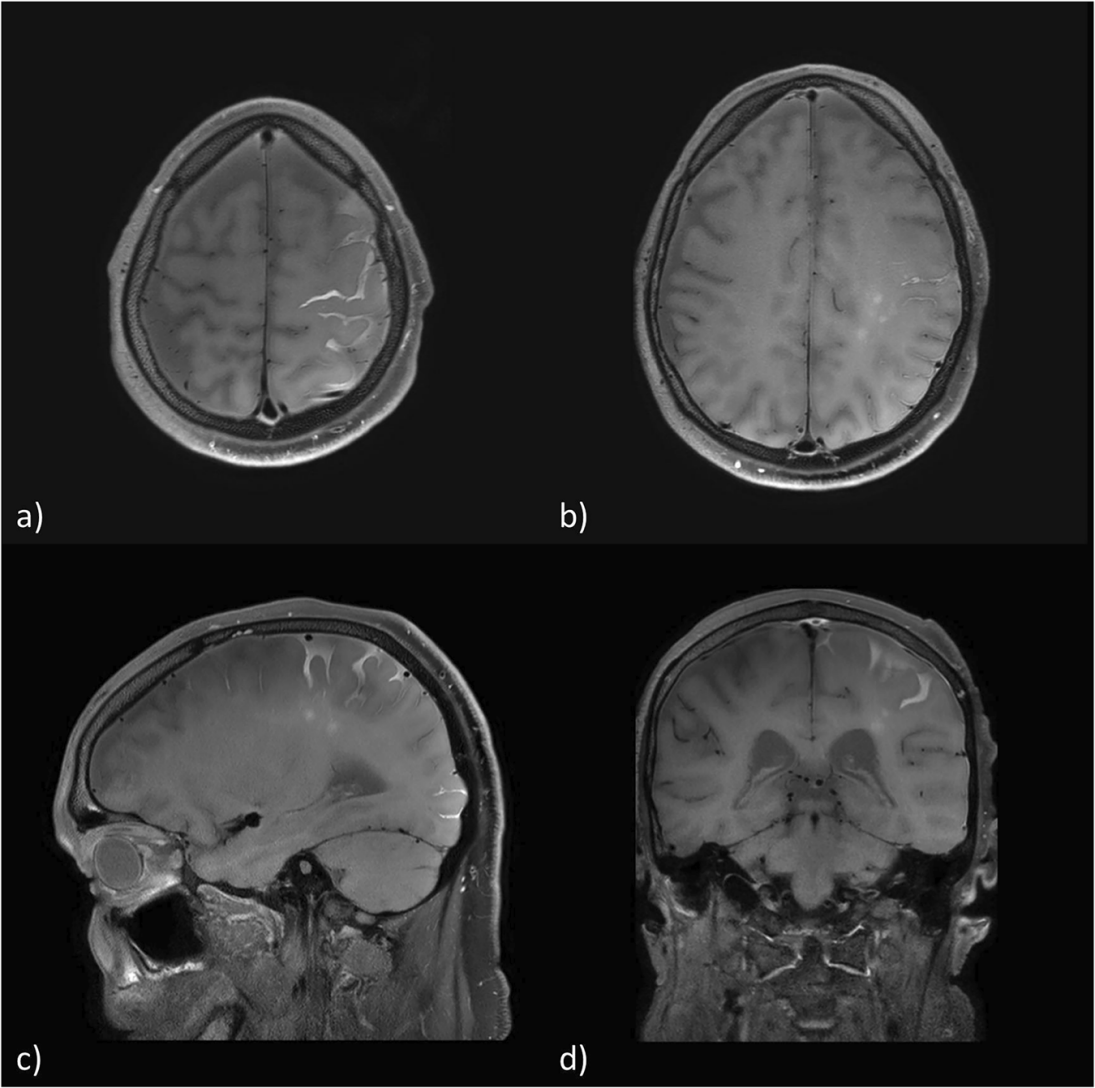
Follow-up MRI examination 3 h after GBCA administration in axial (**a**, **b**), sagittal (**c**) and coronal (**d**) reconstructions of three-dimensional T1-weighted sequence showing a diffuse subarachnoid (**a**, **c**, **d**) and parenchymal (**b**–**d**) GBCA enhancement. GBCA, Gadolinium-based contrast agent; MRI, Magnetic resonance imaging

Regarding the pattern of CE, 29 of 30 patients (96.67%) demonstrated a pial CE (Figs. 2, 3) whereas 17 patients (56.67%) had an additional parenchymal CE which in all cases spatially correlated with the course of the PVS (Fig. 4). One case showed a diffuse subarachnoid CE in a follow-up examination (Fig. 5). In 29 cases the location of the CE correlated or partly correlated with the downstream territory of the probed vessels during a therapeutic DSA or the localization of the pathology in case of a diagnostic panangiography, respectively. Only one patient demonstrated a minimal solely parenchymal CE in the brainstem after a diagnostic panangiography, which did not correlate with the downstream territory of the localization of the aneurysm.

An example is provided in Fig. 4. A 71-year-old patient underwent elective embolization of an unruptured MCA-bifurcation aneurysm with a Woven EndoBridge‒WEB device. The intervention proceeded without any complications. A contrast-enhanced MRI was performed 24 h after intervention (Fig. 4) as part of the standard protocol after aneurysm embolization. Besides a successful embolization, findings were two acute microembolisms in the left centrum semiovale and left superior parietal lobule (Fig. 4f) and a BBB disruption in the MCA territory with pial and parenchymal CE (Fig. 4b, d, e), the last one corresponding to the course of the PVS (Fig. 4c). Due to new neurological symptoms, including paresis of the right hand and right sided facial palsy, the patient underwent MRI again, 3 h later without new GBCA application (Fig. 5). No new infarction or hemorrhage was found. However, contrast accumulation in the subarachnoid space overlying the locations of the BBB disruption detected on the initial scan was now noted. Also, the parenchymal CE initially limited to the course of the PVS was now seen to have diffusely and centrifugally progressed into the brain parenchyma. As the paresis of the arm persisted, a third unenhanced MRI was performed another 24 h later. Here, CE could not be detected anymore. A final MRI after 6 days did not show any relevant additional findings. The patient was discharged with a residual coordination disorder of the right arm. In a clinical follow-up examination 8 months later, no focal neurological deficits were noted by the colleagues of the neurosurgery department.

## Discussion

The present work is the first *in human* demonstration of GBCA transport pathway patterns after iatrogenic BBB disruption due to iodine contrast agent administration. High-resolution 3-T MRI was performed with T1-weighted VISTA sequences known for their high sensitivity to GBCA. This study was able to depict pathways possibly corresponding to those proposed in the initial description of the glymphatic system *in vivo* [1]. All patients with supratentorial BBB disruption showed a pial CE, and only some of them presented a parenchymal CE along the course of the PVS. Thus, it can be hypothesized that there is an individually dependent chronological sequence with first a pial CE followed by a perivascular CE, as the interval between CE and the acquisition of black-blood sequence was always the same.

Yet, the exact localization of the BBB disruption remains unclear. In literature, naturally weak barriers are discussed, compromising the pial arteries and the venous vasculature in the subarachnoid space [23]. Nonetheless, other mechanisms could also explain the observed CE [24]. These include passive CSF-mixing or nonspecific leakage, *e.g*., through the fenestrated capillaries of the choroid plexus [25]. In such instances, the presence of CE would be anticipated in the immediate vicinity. However, the present observations indicate that the CE originates from the pial space on the brain surface. It is hypothesized that tracers enter the brain along pial-glial basement membranes and subsequently exit along the IPAD pathways [24]. One of the key distinctions between the glymphatic and IPAD pathway pertains to the efflux route. Due to the relatively brief interval between GBCA administration and image acquisition, the study is only able to make observations on the influx into the brain parenchyma. Consequently, it is unable to distinguish between the glymphatic and IPAD pathways. Additionally, the current spatial resolution of 3-T MRI seems to be inadequate to discern whether the CE is situated with the pial-glial basement membrane or inside the perivascular space. Another major difference is that the IPAD pathway facilitates a rapid outward drainage of ISF, while the glymphatic system allows for much slower inward flow. The GBCA distribution in the presented case report demonstrates a diffuse CE in the brain parenchyma originating from the perivascular spaces, 4 h after injection.

As stated in the introduction, the SLYM is hypothesized to compartmentalize the subarachnoid space. It has been theorized that the purpose of this mechanism is to facilitate the peri-arterial influx of CSF and to support the unidirectional glymphatic CSF transport [4]. This could explain why the initially observed CE is limited to the immediate vascular environment and is not diffusely distributed in the subarachnoid space. The observation stands in line with a work by Eide and Ringstad [26], who also observed a time-dependent perivascular pattern along the cerebral arteries after intrathecal GBCA administration, suggestive of a compartmentalized subarachnoid space. As the existence of the SLYM is emerging and still under debate, further research, also including repetitive or dynamic MRI examinations after intravenous GBCA administration and using quantitative signal-intensity ratios, is needed to elucidate whether the diffuse subarachnoid CE results from the postulated glymphatic pathways, directly via semi-permeability or loss of SLYM-barrier function or other mechanisms.

The CE seen along the PVS could either result from entrance from the pial space or directly through leakage across the arteriolar vessel wall. To our knowledge, it is not known whether pial arteries are more susceptible to iatrogenic BBB disruption than intraparenchymal arterioles. Nevertheless, current studies mainly depicted CE only inside the PVS of the basal ganglia [11] and were not able to detect it in the white matter, maybe because of the lower spatial resolution of the applied imaging approaches. In addition, the progression pattern described in the case report further supports a correspondence to the described components of the glymphatic system, as the CE initially limited to the PVS was shown to diffusely progress into the adjacent brain parenchyma, possibly signifying the transport from the perivascular to the interstitial compartment. Other studies described BBB opening after focused ultrasound. Here, a mainly perivenous perivascular distribution was described after localizing the CE pattern with the venous architecture on susceptibility-weighted images [15]. Even if the appearance of the CE looked similar with linear and curvilinear hyperintensities, in the present work, however, the observed parenchymal CE corresponded to the course of the PVS in the T2w sequence, which in literature is described to be peri-arterial [27]. The follow-up MRI in the illustrative case report contained an additional susceptibility-weighted imaging sequence where the CE pattern also not correlated with the course of the parenchymal veins. Probable explanation for this phenomenon is that mechanisms and localizations of iatrogenic BBB disruption differ between arterial DSA with iodine contrast agent and focused ultrasound.

Significantly more BBB disruptions were observed after therapeutic compared to diagnostic DSA. This probably results from intervention-related factors such as a higher local contrast agent concentration caused by selective intra-arterial injection and also mechanical impact on the vasculature and vessel wall during distal probing and implantation of embolization materials [28], but possibly also from patient-related factors [29], not further analyzed in this context. In the vast majority of cases, the localization of the BBB disruption correlated with the downstream territory of the probed vessels during DSA. Only two cases with infratentorial BBB disruptions were described in this work, of which in one case the posterior territory was not even probed during a therapeutic DSA. It remains unclear whether BBB and glymphatic system in the infratentorial region differ in composition and functionality from the supratentorial region or if the susceptibility to iatrogenic BBB disruption is lower in the posterior circulation. The structure and potential differences between the supratentorial and infratentorial regions require further extensive research.

The present study found no significant association between either age or sex with the presence of a BBB disruption. Even though this was not expected, this might be due to a selection bias caused by the specific cohort containing patients who underwent DSA for an intracranial aneurysm. It has to be taken into consideration that, besides the presence of localized vascular pathologies, the vast majority of the included patients were healthy and the BBB disruption was iatrogenic. This result might therefore not be transferable to patients with comorbidities possibly affecting BBB integrity. Under these circumstances, sex and age are also relevant factors to be taken into consideration. Only a few patients had a history of relevant neurological or neurosurgical secondary diagnoses with some overlaps: one had a chronic subdural hematoma treated with embolization of the middle meningeal artery, one had an atypical intracerebral hemorrhage, four had a history of subarachnoid hemorrhage, and two had arteriovenous malformations, one of which had been treated in the past. None of these patients underwent angiography in the acute phase of these pathologies.

This work revealed significant associations between the presence of BBB disruptions and the time between DSA and MRI. However, these results might be biased by the fact that therapeutic interventions were generally longer, and the time between DSA and MRI was much shorter after diagnostic DSA. The time point of the MRI examination after iatrogenic BBB disruption should be standardized in future prospective work with potentially repetitive examinations.

The present study showed a significant association between the presence of BBB disruption and the occurrence of embolisms, defined as diffusion restriction in DWI. However, this result needs to be treated with caution, as this might be confounded by a potential correlation with intervention duration itself. The link to neurological symptoms remains a topic of discussion in the literature, from sources describing BBB disruptions as a minor finding [30] to sources significantly associating BBB disruptions with neurological deficits [22]. The results of the present work support the thesis of BBB disruptions being a minor finding that is usually not accompanied by neurological symptoms. As described above, clinical symptoms were documented for 9 patients after DSA, 8 of whom had a BBB disruption. Most symptoms were nonspecific, did not exceed headache or nausea and did not include any focal neurological deficits. Only two patients developed focal neurological deficits. These symptoms could in both cases be explained by the presence and localization of embolism in the according brain areas. This might suggest that in studies using postinterventional CT imaging, the sensitivity of this modality is not high enough to depict small embolisms, which could also explain focal neurological deficits.

This work also has some limitations. Besides the relatively small number of patients, due to the retrospective design, in most cases, only one MRI examination after DSA was performed. Two patients received a follow-up MRI, of which only one included a BB sequence, as described in the illustrative case. Also here, it remains unclear if the diffuse subarachnoid CE evolves directly from the pial CE, possibly due to semi-permeability or loss of SLYM-barrier function or if the contrast agent took its way from the peri-arterial PVS through the glymphatic system and via perivenous PVS to the subarachnoid space. Additionally, the seen ventricular CE might derive from GBCA penetration through the fenestrated vasculature of the choroid plexus into the ventricular CSF [10]. With the present study design, it is not possible to draw any conclusions about the cellular properties of either the intact and disrupted BBB or the postulated SLYM, which makes further research necessary. Presence and extent of BBB disruption depend on the time interval between DSA and MRI, which varies from 1 to 26 h in the present study. This limitation should be addressed in further studies with fixed and/or serial time intervals.

In the present work, the cohort is restricted to patients undergoing DSA for aneurysm-related reasons, limiting generalizability. However, this relatively healthy cohort was chosen to minimize the effects of possible circumstances of BBB weakness. Furthermore, no correlation between the aneurysm size, used device and the presence of BBB disruption was performed. However, it is explicitly not the aim of this study to analyze technical aspects of the intervention with regard to the presence of BBB disruptions, rather than the patterns of BBB disruption. Former studies already showed that there is no correlation between the used device and reversible contrast agent accumulation [22].

In conclusion, the present work shows that GBCA can be traced along the pial compartment and PVS after crossing the BBB with a 3D high-resolution T1-weighted VISTA sequence, probably visualizing components of the proposed glymphatic drainage pathway. This work can be understood as a hypothesis-generating pilot project. In this study, an iodine contrast agent was used for iatrogenic BBB disruption to follow the GBCA-enhancement pattern. Further and prospective work with dynamic/serial examinations is needed to better characterize the exact pathways and velocity of GBCA after crossing a non-intact BBB and their relationship to the glymphatic system or alternative pathways, such as the IPAD, and to determine the components of the postulated SLYM. However, these imaging changes visualized by a 3D high-resolution T1-weighted VISTA sequence can aid in further understanding of tracer pathways in circumstances with impaired BBB or glymphatic dysfunction.

## Data Availability

The data used in this study are not publicly available due to privacy reasons. Access to the data can be requested from the corresponding author under specific conditions and will be provided upon reasonable request, subject to ethical approval and relevant data sharing agreements.

## Abbreviations

3D: Three-dimensional
BBB: Blood-brain barrier
CE: Contrast enhancement
CNS: Central nervous system
CSF: Cerebrospinal fluid
CT: Computed tomography
DSA: Digital subtraction angiography
DWI: Diffusion-weighted imaging
GBCA: Gadolinium-based contrast agent
IPAD: Intramural peri-arterial drainage
ISF: Interstitial fluid
MCA: Middle cerebral artery
MRI: Magnetic resonance imaging
PVS: Perivascular space(s)
SLYM: Subarachnoid lymphatic-like membrane
VISTA: Volume-isotropic turbo spin-echo acquisition
